# Higher Diplomas

**Published:** 1909-09-04

**Authors:** 


### HIGHER DIPLOMAS.

*The Royal College of Physicians of London* grants its membership after examination to graduates in medicine of recognised Universities, or to licentiates of the College, being above the age of twenty-five years, who do not engage in trade, do not dispense medicine, and who do not practise in partnership. The examination, which is held in January, April, July, and October, is partly written and partly oral. The fee for the membership is £42, but if the candidate is a licentiate the fee is the difference between what he has already paid and £42. In either case £6 6s. is paid before examination. The fellowship is elective.

*The Royal College of Physicians of Edinburgh* admits to its membership licentiates or graduates over twenty-four years of age after examination in medicine and therapeutics and one or more of the following subjects selected by the candidate: (1) One or more departments of medicine specially professed, (2) psychological medicine, (3) general pathology and morbid anatomy, (4) medical jurisprudence, (5) public health, (6) midwifery, (7) diseases of women. A candidate forty years of age and ten years in practice *may be excused* any part of or all the examination by the Council. The fee to be paid by a licentiate is fifteen guineas, by others thirty-five guineas. A member of three years' standing may be elected a Fellow. The fee is £64 18s.

*The Royal College of Surgeons of Edinburgh.*—The Fellowship is granted, after examination, to any registered practitioner who is twenty-five years of age and has been in practice for two years. The examinations are written, oral, and practical. The fee is £30 to those who hold the diploma of licentiate of the College, and £45 to others (no stamp duty is payable on the diploma).

*The Faculty of Physicians and Surgeons of Glasgow.*—Registered practitioners are admitted to the Fellowship by examination and by subsequent election. Four examinations are held annually (January, April, July, October). Fourteen days' notice must be given. The fee is £30 unless the candidate desires to qualify to hold office, when it is £50. In the case of a licentiate of the faculty, £25 and £15 respectively.

*The Royal College of Surgeons of Ireland* grants the Fellowship after examination. If the candidate is of less than ten years' standing he is examined (at the end of his third winter course of dissections) in anatomy, including dissections, physiology, and histology. For the Final, surgery, including clinical and operative surgery and surgical pathology, are the subjects. If of over ten years' standing the subjects are surgical anatomy, surgery, and surgical pathology.

